# Critical Review of Brown and Thomas “The First New Zealanders? An Alternative Interpretation of the Stable Isotope Data from Wairau Bar”

**DOI:** 10.1371/journal.pone.0137616

**Published:** 2015-10-28

**Authors:** Rebecca L. Kinaston, Ben Shaw, Andrew R. Gray, Richard K. Walter, Chris Jacomb, Emma Brooks, Sian E. Halcrow, Hallie R. Buckley

**Affiliations:** 1 Department of Anatomy, Otago School of Medical Sciences, University of Otago, Dunedin, New Zealand; 2 School of Biological, Earth and Environmental Sciences, University of New South Wales, Sydney, Australia; 3 Department of Preventative and Social Medicine, University of Otago, Dunedin, New Zealand; 4 Department of Anthropology and Archaeology, University of Otago, Dunedin, New Zealand; 5 Southern Pacific Archaeological Research, Department of Anthropology and Archaeology, University of Otago, Dunedin, New Zealand; University of South Carolina, UNITED STATES

## Introduction

This formal comment has been written in response to Brown and Thomas’s article in the current issue of *PLoS ONE* entitled “The first New Zealanders? An alternative interpretation of the stable isotope data from Wairau Bar”. In their article, Brown and Thomas attempt to reanalyse and re-interpret the stable isotope (δ^13^C and δ^15^N) and radiogenic isotope (^87^Sr/^86^Sr) data originally presented in Kinaston *et al*. [1] “The first New Zealanders: patterns of diet and mobility revealed through isotope analysis.” *PLoS ONE* 8(5): e64580. Brown and Thomas (p. 2) were prompted to undertake their ‘critical review’ of our study as a result of a disagreement with the groupings of burials, and state “ultimately, these divisions introduce bias, which leads to conclusions that we feel are not supported by the available data.”

The criticisms of Brown and Thomas are not valid. There are three major flaws in the analyses and interpretations, and their findings do not add anything meaningful to the interpretations we already presented. The first flaw is a misunderstanding by Brown and Thomas about the differences between the two approaches and the logic of the argument we set out. The second relates to their treatment of stable isotope data, especially their new dietary interpretations. The third pertains to the way they have used the biologically available ^87^Sr/^86^Sr baseline (from dog enamel) to interpret the human ^87^Sr/^86^Sr ratios and their use of a ranked ordering of the human ^87^Sr/^86^Sr ratios. We present our detailed responses in relation to each of these flaws as follows, in addition to comments regarding more minor inconsistencies in their analysis and interpretations.

Brown and Thomas argue that we were wrong in the way they grouped burials at Wairau Bar and thus in the way they constructed analytical units. They attempt to demonstrate this by taking the isotope data from Kinaston *et al*. and running it through a number of cluster analyses while “treating the isotope data independently from cultural or biological factors” (Brown and Thomas). Brown and Thomas are not able to replicate the existence of Group 1 by clustering the stable isotope values and ^87^Sr/^86^Sr ratios and this, they believe, undermines our findings.

This criticism misses the fundamental point of Kinaston *et al*. Group 1 (Burials 1–7) is an independently defined group based on a range of archaeological and biological factors. The grouping was first introduced as a concept by Duff (page 61 in [2]) who suggested that “…burials 1–7, appear to have been the exclusive resting place of men of superior rank”. The burials 1–7 grouping was further validated by Anderson (Table 9.1 in [3])who grouped all the burials according to the presence or absence of moa eggs, moa bone elements, artefact types, and material used for artefact manufacture. Brown and Thomas dismiss the differences in grave good type and number as simply being a result of taphonomic bias, providing a convenient means to disregard the evidence that is actually available; specifically that “there is a ratio of approximately 5:1 in favor of Group 1 in terms of mean numbers of grave goods” (page 2 in [1]). There are also stratigraphic grounds justifying this treatment of burial groups which were first noted by Owen Wilkes (summarized in [3]). Most recently, Buckley *et al*. [4] re-evaluated the health indicators of the entire Wairau Bar burial population and discussed the data in reference to these groupings. They found that oral health, which reflects aspects of diet, further reinforces the identification of burials 1–7 as a group.

Thus we. do not need to demonstrate the existence of Group 1; it is already well attested in the literature [3,4]. The logic of our approach is as follows:

1. There is a group of burials (1–7) at Wairau Bar, which for reasons that include spatial, stratigraphic, material culture and other factors (some of which were outlined in the text and others cited below), have proven to stand out from the others in interesting ways.
2. Can we add knowledge about the diet and origins of this group through the application of new analytical methods?

## Explaining Exploratory versus Confirmatory Analyses

To take a data-driven exploratory approach such as cluster analysis, as Brown and Thomas have done, would not have been appropriate in our study because this would address a very different question. They are effectively looking for ‘patterns’ within the data based on isotope values, while we were examining how isotope values were distributed across the population given the already accepted groupings. Data-driven exploratory approaches can be very useful for generating new hypotheses, such as “are there groups”, but are much less useful for examining whether or not the new, in this case the isotope, data are consistent with an existing model. Cluster analysis in its conventional (non-Bayesian) form fails to make direct use of this existing knowledge in the modelling process. In summary, the approach Brown and Thomas used would have been entirely appropriate if we were interested in the question “are there possibly different groups amongst the burials?” but are not at all appropriate for addressing our question about whether Group 1 was distinct from the rest of the sample in terms of diet and origins.

Furthermore, it is important to note that when hierarchical cluster analysis is performed in an agglomerative fashion, there are implications from the choice of linkage method, and different linkage methods can produce very different clusters. With single linkage, there is the well-known problem of observations combining in chains, which can produce ultimately meaningless clusters. Other approaches, including Ward’s linkage as used in Brown and Thomas’s article, are often not stable in the presence of a small number of changes to the data set and, as Blashfield and Aldenderfer (page 450 in [5]) noted,“stability is an important property of any classification in that stable groups are more likely to represent ‘natural’ groups in the data than those which disappear when cases are reordered or a few cases are omitted”. Such problems are especially common in small data sets, as is the case here. We note that Brown and Thomas do not present sensitivity analyses to explore the robustness of their findings.

The hypothesis-driven confirmatory approach that we used is more appropriate for our study because it applies existing knowledge about the burials and how they had been grouped, and then develops hypotheses about those groups in terms of the isotope data. We tested these hypotheses using statistical tests of means, including t-tests and regression models; tests of variances, in particular Levene’s test; and correlations. This approach allowed us to ask the question of interest to us, specifically: *Are there differences between the burials in Group 1 and the remainder of the burials (Group 2/3) that cannot be readily attributed to chance*?

It is not surprising that the two approaches result in different interpretations of the data as the goals are clearly different. By way of example, suppose we were to perform both a test for difference in means and an exploratory cluster analysis on heights from a sample of individuals suspected to be from two distinct populations which are known to have different mean heights but whose height distributions overlap. It would not be at all surprising if, despite finding that the mean heights for the two groups differed statistically significantly as expected, we also found individuals from the two different populations ended up included in the same clusters. In fact, it would be remarkable if the resulting clusters exactly reflected the underlying populations, especially with a mixture of men and women in the sample. If there was any overlap between the isotope values for the underlying groups, similar issues could readily arise in a cluster analysis of that data also.

## Flaws in the Interpretation of the Stable Isotope Data

Having made the point that Brown and Thomas are asking different questions of the isotope data we now consider their interpretation of those data that demonstrate their lack of understanding of stable isotope analysis and the results presented in Kinaston *et al*.

### 1. A misunderstanding of the dietary interpretations presented by Kinaston *et al*.

First, Brown and Thomas misunderstand the way in which we interpret dietary differences between the two groups. Brown and Thomas rely on the assertion that the statistical models used by us show that “no reliable significant difference has been found between these groups to suggest that they are distinct populations”. However, as we explain, the significant difference in the *variability* of δ^13^C values and the differences in the correlations of δ^13^C and δ^15^N values between the groups demonstrate that the dietary *patterns* are different (pages 6–7 in [1]). These results, and the range in values observed for each group, indicate that the people from Group 2/3 were eating marine and terrestrial foods from varying trophic levels, whereas the individuals in Group 1 were consuming a diet that was *less variable* and included marine and terrestrial resources from similar trophic levels or a major protein source. This is very clear from a simple plot of the carbon and nitrogen stable isotope ratios (figure 4 in [1]) in reference to the dietary baseline. This is not apparent in Brown and Thomas’s cluster diagram because, as explained above, cluster analysis is not the appropriate method to use with the hypothesis being tested. It is important to realize that different diets can produce isotope values that appear similar and therefore it is necessary to use other statistical tests (discussed above) that might inform about other dietary patterns.

Brown and Thomas disregard the results of the differences in variability because they contest the original division of the groups and dismiss the results “as an artefact of classification” and state “that a discrete cluster of burials exhibits less dietary variability than the site as a whole is unsurprising”. However, if Group 1 were simply a subset of the wider population, as Brown and Thomas suggest, then this group could be expected to have a range of stable isotope values that was more similar to the rest of the population (which is relatively large). This is not the case, and Group 1 display a restricted diet in comparison to Group 2/3. This is meaningful precisely because burials 1–7 have been shown on the basis of independent data to comprise an archaeologically distinct cluster. It should be noted that Brown and Thomas do not comment on the differences in correlations of δ^13^C and δ^15^N values between the groups, which also show important differences in the dietary patterns (pages 6–7 in [1]). Yet again, it is important to stress that Group 1 was accepted by previous researchers as a valid analytical unit regardless of their isotope values.

### 2. Problems with the interpretation of the perceived sex differences in diet at Wairau Bar

The second flaw in the stable isotope analysis presented by Brown and Thomas is the way in which they interpret perceived sex differences in diet at the site. Brown and Thomas claim that the explanation for variability in the isotope data can be better explained by differences in diet between males and females. They demonstrate this using hierarchical agglomerative cluster analysis of the stable isotope values and isolate four females, one male and one individual of unknown sex as falling within a distinct cluster (Brown and Thomas, Cluster One of their Fig 2). The predominance of females in this cluster leads them to the conclusion that sex is the determining variable. For the overall sample a significant difference was found between the sexes for δ^15^N values, but not the δ^13^C values, although no significant differences were found when the males and females from their Cluster Two were compared alone. The four females with the highest δ^15^N values in their Cluster One are therefore the likely reason for raising the female mean of the whole population. Brown and Thomas do not note that when multivariate modeling is used, the significant difference in δ^15^N values between the sexes was not found after adjusting for age (*p* = 0.07) although it is later stated that “visual assessment of Figs 4–7 suggests that Cluster One may also be characterised by older individuals with fewer grave goods”, suggesting that age may have had an influence on their results. Importantly, only four of the ten females in the whole Wairau sample (in addition to the male and individual of indeterminate sex) are identified by their cluster analyses. Therefore, their cluster analyses do not clearly identify sex differences in diet for the whole sample. A more parsimonious interpretation of their data would be that the large variation in diet is likely a result of the different landscapes that these highly mobile people occupied (as ascertained from the ^87^Sr/^86^Sr ratios) and obtained food from, which we suggested (page 9 in [1]) for the variability observed in Group 2/3.

Brown and Thomas interpret the higher δ^13^C and δ^15^N values of their Cluster One individuals as representing a diet containing a higher proportion of marine foods, which is correct. However, the stable isotope values of the Cluster One individuals are representative of higher trophic level fish and/or marine mammals. They focus on the four females in Cluster One, and do not further elaborate on the diet of the male and individual of indeterminate sex. They explain the dietary differences of these four women as possibly resulting from gendered differences in food procurement activities, such as collecting estuarine and inshore organisms but, if that were the case, they would have exhibited *lower* nitrogen stable isotope ratios than those observed (for dietary baseline information on inshore organisms see [6,7]). This also contradicts the assumption that men were hunting and eating foods that were “energy rich but less reliable”, including marine mammals and higher trophic level fish in prehistoric New Zealand. Moreover, Brown and Thomas state that their new findings of sex differences in diet in the Wairau Bar sample are a similar dietary trend to that found at the Teouma site, when in fact the opposite result was found at Teouma [6]. At Teouma, the higher δ^15^N values of the male individuals compared to females indicate that men were consuming foods from higher trophic levels compared with the females, most likely meat, and these differences were statistically significant [6]. The dietary patterns observed at the Teouma site fit better with Brown and Thomas’s interpretation of the specific gendered differences in food procurement observed in the Pacific islands, but may also represent preferential food allocation of foods considered ‘high-status’ in the region, such as meat and greasy/fatty foods.

Another point is that there have been several attempts to determine the sex of the individuals interred at Wairau Bar (e.g. [2, 8]), the most recent estimates by an experienced team of experts using the most up to date methods was presented in Buckley *et al*. [4]. Brown and Thomas do not state which source of sex estimates they used in their critique.

## Flaws in the Interpretation of the ^87^Sr/^86^Sr Ratios

In regard to the mobility aspect of our study, Brown and Thomas argue that “*it would be more accurate according to their standard*, *to argue that we can be 95% sure that 20 individuals are non-local*.” This misinterprets the meaning of a 95% reference range. This is a range which 95% of values are expected to fall within and 5% of observed values would be expected to fall outside of this range even if they belong to the same population. Values which do not fall within that range cannot be said to be 95% likely to be non-local as this depends on the typical values for the non-local population. However, if the estimate of the mean and standard deviation were accurate, we could distinguish between values as consistent with or unlikely under their assumption. More important here, however, is the limited sample size available to construct this range because of the imprecise estimates of both the mean and, particularly, the standard deviation. The ^87^Sr/^86^Sr analysis of the dogs’ teeth is used only as a first order indication of what the local *biologically available* strontium isotope range (as opposed to geological strontium) would be in the vicinity of the site, providing that the dogs lived in the immediate Wairau Bar environment and consumed locally obtained food. Given the very narrow range of ^87^Sr/^86^Sr ratios for the dogs’ teeth this was likely the case. However, as only five dogs’ teeth were available for sampling, the application of a ±2 SD range on such a small sample cannot adequately define the local ^87^Sr/^86^Sr isotope variation. The problem, as we discussed and reiterated by Brown and Thomas, is the lack of information on the biologically available strontium in the wider Wairau region. In the absence of such a baseline, we have instead used the ±2 SD range as a basic measure to establish how much variation is observed in the human sample relative to an estimate of a local signature. We therefore do not suggest that Group 2/3 are all local given the preliminary nature of the data, as we state: “….*the large range in Group 2/3* ^*87*^ *Sr/* ^*86*^ *Sr ratios*, *many of which are greater than two standard deviations from the average dog* ^*87*^ *Sr/* ^*86*^ *Sr ratios*, *could be interpreted in a number of ways”*(page 7 in [1]). That is to say, they could also include non-local individuals. In the context of isotopic mobility/migration studies, this does not mean non-local immigrants came from a single point on the landscape (especially if people were highly mobile), but they could have come from any number of locations across a geological landscape and therefore the range of values we found is to be expected.

**Fig 1:**
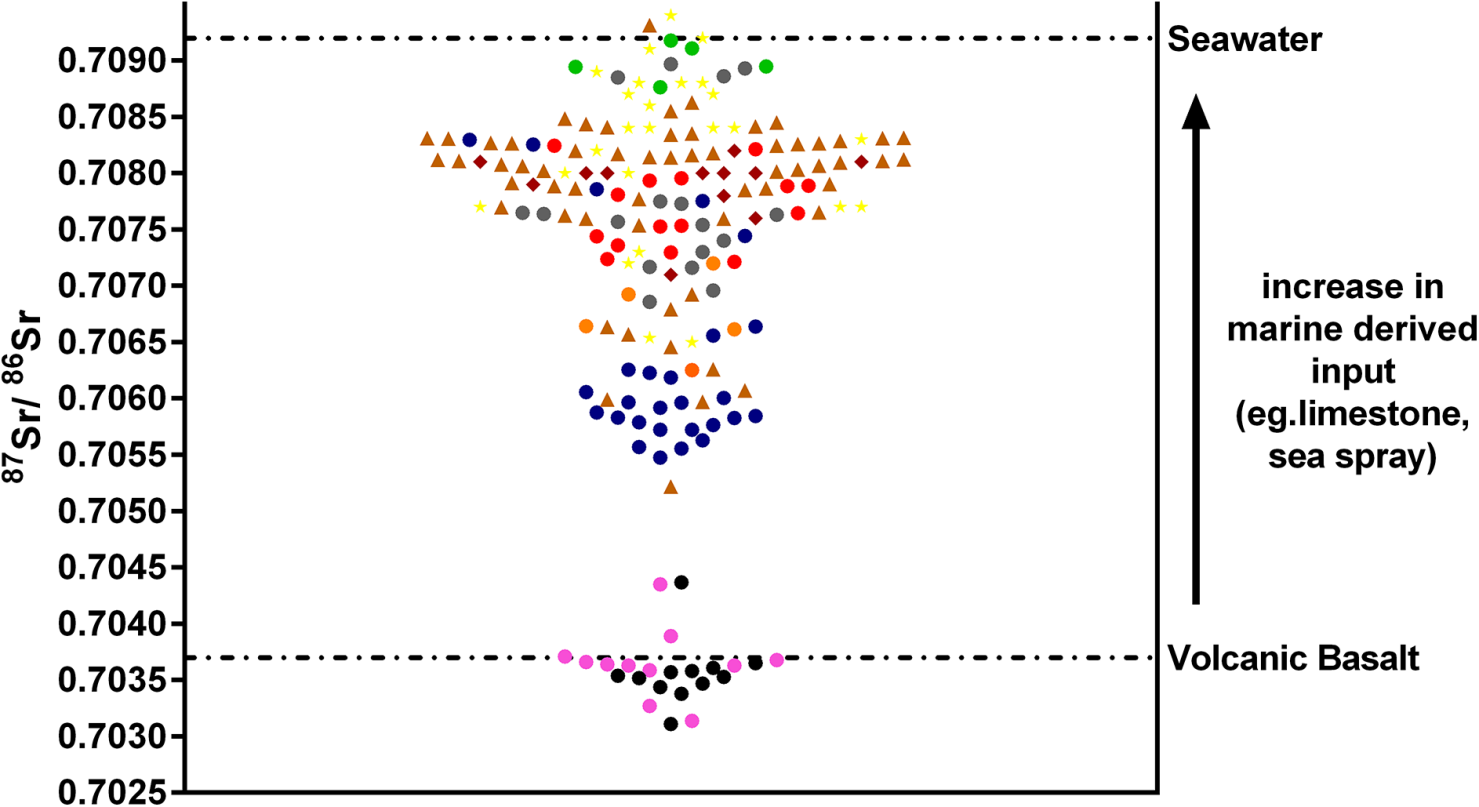
^87^Sr/^86^Sr ratios for human dental enamel samples from the Pacific Islands. Averaged values for volcanic basalt and seawater are provided, from Faure [13]. Samples included in order of size: Fiji (N = 61, brown triangles; [14]), Nebira (N = 27, blue circles; [15]), Teouma (N = 17, grey circles; [16]), Wairau Bar (N = 24, yellow stars; [1]), Watom (N = 15, red circles; [17]), Mangaia (N = 12, maroon diamonds; [1]), Tongatapu (N = 12, black circles; [18]), Sohano (N = 11, pink circles; [18]), Anir Islands (N = 5, orange circles; [19]), Tanga Islands (N = 5, green circles; [17]).

Secondly, Brown and Thomas compare the structure of the Wairau data (n = 24 humans and n = 5 dogs) to that of Teouma (n = 17 humans; no baseline ^87^Sr/^86^Sr was analysed) by ranking the data from low to high values and note that there is a clearer distinction between those individuals from Teouma interpreted as local and non-local compared with the Wairau sample. In doing so, Brown and Thomas demonstrate they are not aware of the large body of literature that has used ^87^Sr/^86^Sr ratios for human mobility studies. Strontium isotope analysis of prehistoric human enamel samples from Southeast Asia (e.g. [9]), South America (e.g. [10]) and Europe (e.g. [11]) show a more or less continuous range of ^87^Sr/^86^Sr values and not a series of clear separations between local and non-local individuals. In continental regions such as these, the range exhibited in tooth enamel from a single site is very large, where individuals may have come from any number of localities, or had derived isotopic input through their diet from several localities or sources (i.e. marine vs terrestrial). The division between local and non-local is often difficult to determine categorically. For example, in the Pacific island context, the human ^87^Sr/^86^Sr isotopic data from tooth enamel in all the studies conducted in the Pacific islands to date show a similar continuous distribution to that in other regions and one that covers a large range (Fig 1) [12–18]. Therefore, if the Teouma analysis was conducted on a larger sample, it is likely that the gap between local and non-local would become less distinct, with some individuals exhibiting ^87^Sr/^86^Sr ratios intermediate between the two groups. The same sharp division between local and non-local as seen in the Teouma sample would not be expected for Wairau Bar because Wairau Bar is situated in the South Island of New Zealand, a region that exhibits an extraordinary range of ^87^Sr/^86^Sr ratios several magnitudes larger than the andesitic islands of the western Pacific and the islands of Tropical East Polynesia (TEP) reflective of a *continental island’s* geological landscape.

Finally, Brown and Thomas state that *‘whilst we agree with Kinaston et al*. *that it is likely the large variation in* ^*87*^ *Sr/* ^*86*^ *Sr ratios exhibited by the Wairau Bar sample is indicative of a very mobile population*, *we believe we do not yet know enough to make any secure claims about origins*.*’* To clarify, we do not identify any origins for the suggested non-local individuals at Wairau Bar. Indeed, we (page 8 in [1]) highlighted that the large overlap of ^87^Sr/^86^Sr ratios between geological regions within New Zealand and the TEP island geological strontium as a limitation for determining origins of migrants with any level of certainty and stating that the ^87^Sr/^86^Sr ratios of the potential non-local individuals “could represent a wide variety of potential bedrock sources within New Zealand and abroad…”.

Importantly, the Group 1 individuals all display similarly low ^87^Sr/^86^Sr ratios, whereas the rest of the population display highly variable ^87^Sr/^86^Sr ratios. In support of this, the dietary isotopes show a similar trend (i.e. low variability), even though the isotope from bone collagen is representative of a different period of life (~ last 10 years of diet before death) compared with the ^87^Sr/^86^Sr ratios of tooth enamel (representative of the place of childhood residency). The PCA analysis presented in Kinaston *et al*. (Fig 6 in [1]), provides further support that the Group 1 are distinct from the rest of the sample in respect to dietary and mobility patterns.

Therefore, in their discussion of the ^87^Sr/^86^Sr isotope results Brown and Thomas provide no clarity or insight into the interpretations of these data presented by Kinaston *et al*. In this part of the world where isotopic studies of this nature are in their infancy, further clarity can only be gained by pursuing ^87^Sr/^86^Sr isotopic analysis on human, animal and plant samples in other parts of New Zealand and in TEP, a point which was stated clearly in our conclusion.

## Further Comments

Brown and Thomas present no new data, but use various statistical techniques to look for differences between individuals on the basis of anything but group membership. There are numerous ways to interpret isotope data and palaeodietary and palaeomobility studies require a nuanced understanding of isotope science and other lines of evidence that can aid in a meaningful understanding of isotope values with regards to diet and movement in the past. Kinaston *et al*. not only interpreted the data within the context of the available archaeological information but also constructed the first comprehensive dietary baseline available for the Wairau Bar site which was an integral part of our dietary interpretations, a point not mentioned by Brown and Thomas.

Brown and Thomas’s re-analyses of the raw data presented in Kinaston *et al*. using cluster algorithms are not interpreted with the full array of contextual information of the Wairau Bar site, their palaeodietary findings are inconsistent with the dietary evidence from other New Zealand and Pacific island sites, and do not take into account the ethnographic information regarding sexual division of labour and food distribution in these regions. Brown and Thomas state that “the observation of sex-based differences in food consumption due to either division of labour or differential status provides interesting insight into the social structure of early period sites”, yet they do not elaborate on what this might mean in the context of the early colonization of New Zealand, including the Wairau Bar site.

Although their techniques provide different ways of exploring these types of data and may well provide some new insights, as demonstrated above, they have not used appropriate ways of testing the questions raised by Kinaston *et al*. nor do they in any way invalidate our results. By “Treating the isotope data independently of cultural and biological factors” Brown and Thomas effectively abandon other lines of evidence that aid in the interpretation of these data. In reference to human mobility studies, Pollard’s (page 637 in [19]) the following statement is equally valid for any isotope study: ‘‘it is important to consider first and foremost the isotope data in a wider archaeological context…A key test is to ask whether the rest of the archaeological evidence supports or refutes such observations”. We believe Kinaston *et al*. exemplifies this more holisitic model of investigating the past.
